# Estimulação Auricular do Nervo Vago na Insuficiência Cardíaca: Análise Crítica e Perspectivas Futuras

**DOI:** 10.36660/abc.20230298

**Published:** 2023-06-07

**Authors:** Denise Tessariol Hachul

**Affiliations:** 1 Hospital das Clínicas Faculdade de Medicina Universidade de São Paulo São Paulo SP Brasil Instituto do Coração do Hospital das Clínicas da Faculdade de Medicina da Universidade de São Paulo , São Paulo , SP – Brasil

**Keywords:** Estimulação Auricular/métodos, Nervo Vago, Epilepsia/prevenção e controle, Insuficiência Cardíaca, Estimulação do Nervo Vago/métodos, Estimulação Elétrica Nervosa Transcutânea/métodos

O termo VAGO, proveniente do latim “vagar”, foi escolhido para denominar o nervo craniano com a mais complexa diversidade de funções e que afeta inúmeros processos fisiológicos, como a regulação autonômica, imunológica, cardiovascular, gastrointestinal, respiratória e endócrina. ^1^

O nervo vago é composto por 20% de fibras eferentes e 80% de fibras aferentes, que fazem conexões recíprocas entre o cérebro e nossos órgãos. As fibras aferentes transmitem informações sensoriais ascendentes e terminam em quatro núcleos localizados no bulbo cerebral, incluindo o núcleo do trato solitário (NTS). Suas funções eferentes incluem o envio de sinais colinérgicos parassimpáticos de núcleos cerebrais para os órgãos-alvo. ^1 , 2^

A estimulação invasiva do nervo vago (ENV) está estabelecida como terapia para epilepsias refratárias há aproximadamente 20 anos. Pelo fato de dispor de uma entrada única para o cérebro, sua eletroestimulação promove um acesso modulador sobre determinadas áreas subcorticais encefálicas. ^3 - 6^

Na década de 30 se sabia que a ENV causava alterações no eletroencefalograma (EEG). Em 1988, foi implantado o primeiro dispositivo e em 1997 foi aprovado pelo FDA para o tratamento de epilepsia refratária, após demonstrações de redução do número e gravidade das crises, em pacientes não responsivos aos tratamentos farmacológico e cirúrgico. ^5 , 6^

O então “estimulador” vagal tem sido implantado por meio de uma cirurgia minimamente invasiva, com inserção de um eletrodo ao redor do nervo vago cervical esquerdo e de um gerador de corrente na região intraclavicular, semelhante ao marca-passo cardíaco artificial. ^4^ A estimulação do lado esquerdo é preferida por reduzir o risco de bradicardia ou assistolia, que pode ser induzida pela estimulação do vago cervical direito.

A ENV pode provocar uma gama de efeitos colaterais: desde rouquidão, disfagia e dor local (pela sua proximidade o nervo glossofaríngeo), até súbitos bloqueios atrioventriculares, por seu efeito eferente sobre o nó sinusal e atrioventricular, mas o aprimoramento técnico e ajustes dos parâmetros de estimulação tornaram compensador o balanço risco/benefício, especialmente por reduzir a taxa de mortalidade em pacientes portadores de epilepsias intratáveis. ^4 - 6^

Apesar de numerosos estudos demonstrando sua eficácia, o mecanismo pelo qual a ENV controla a epilepsia ainda não foi totalmente desvendado. Há hipóteses de que atue na redução da excitabilidade cerebral ^1^ e por ação anti-inflamatória, já que recentemente se demonstrou o papel da inflamação na ocorrência de crises epilépticas.

Após o sucesso da terapia para epilepsia, os efeitos da ENV passaram a ser mais estudados e aplicações clínicas começaram a ser experimentadas para uma gama de doenças, ^7 - 12^ como enxaqueca, obesidade, depressão, doenças inflamatórias intestinais e insuficiência cardíaca (IC). ^13 - 15^

No contexto da IC, ainda utilizando dispositivos implantáveis, dois importantes estudos clínicos foram realizados. O NECTAR-HF demonstrou melhora estatisticamente significante na qualidade de vida e na classe funcional (NYHA) no grupo submetido à intervenção, mas não nos parâmetros ecocardiográficos e no tônus autonômico. No INOVATE-HF, os pacientes que receberam dispositivo implantável ou tratamento médico convencional, não apresentaram diferença nos desfechos primários (morte por todas as causas, piora da insuficiência cardíaca e no volume sistólico final do ventrículo esquerdo). Mas houve melhora da qualidade de vida e no resultado do teste de caminhada de 6 minutos no grupo estimulado. ^14 - 16^

Pelo seu caráter invasivo, efeitos colaterais e complicações inerentes à cirurgia, modalidades não invasivas de ENV passaram a ser desenvolvidas, particularmente a estimulação transauricular. ^1 , 2 , 17^

O ramo auricular do nervo vago pode influenciar o sistema cardiovascular indiretamente, por vias aferentes que atingem o núcleo do trato solitário (NTS), cujos neurônios se projetam para neurônios vagais centrais eferentes cardioinibitórios. Esses, por sua vez, propagam a atividade parassimpática para o nó sinoatrial e átrio ventricular. O NTS, por meio de outras vias, também inibe o impulso excitatório para os neurônios pré-ganglionares simpáticos da medula espinhal. Essa inibição diminui a atividade simpática no sistema cardiovascular. ^18 , 19^

Embora potencialmente eficaz e menos invasiva, os protocolos propostos de estimulação auricular e seus resultados apresentam grandes variações. ^2 , 20 , 21^ A região engloba outros nervos auriculares que podem ser estimulados inadvertidamente.

A estimulação de diferentes nervos auriculares pode induzir efeitos cerebrais e periféricos diversos, que afetam a reprodutibilidade do método. ^2 , 22^ Múltiplos reflexos podem ser acionados, tais como o reflexo de tosse de Arnold e do lacrimejamento, cefaleia, irritação da pele local, tonturas e mesmo síncopes.

O artigo publicado nos Arquivos Brasileiros de Cardiologia, ^23^ avaliou prospectivamente 2 grupos de pacientes com IC compensada. No grupo intervenção, utilizaram um eletrodo transcutâneo na concha superior e o outro no lóbulo da orelha direita, com o objetivo de estimular terminações do vago, mas também do grande nervo auricular do lóbulo, por facilidade técnica e uniformização do protocolo. No grupo *sham* , ambos eletrodos foram alocados no lóbulo auricular direito. O tempo de intervenção foi empiricamente determinado, assim como a duração do tratamento.

O grupo intervenção apresentava idade mais elevada e maior índice rMSSD e o grupo *sham* tinha melhor índice de qualidade de vida e melhor classe funcional, no momento do recrutamento. Em ambos os grupos houve uma melhora do desempenho no TC6min. No grupo intervenção a melhora foi estatisticamente significativa, mas ainda assim, a performance foi inferior à do grupo *sham.*

Observou-se melhora na qualidade de vida no grupo intervenção, sem diferença no grupo controle, cuja qualidade de vida era superior antes da intervenção.

A variabilidade da frequência cardíaca é um método consagrado há anos para avaliar o tônus vagal, uma variável considerada cardioprotetora, associada a outras variáveis clínicas e laboratoriais. ^24 - 26^

Nota-se que os valores de rMSSD eram maiores antes das intervenções no grupo *sham* e não se observou diferença significante entre os grupos após o tratamento. O único parâmetro que apresentou melhora após estimulação foi o SDNN. No entanto, é difícil avaliar o valor de um parâmetro isoladamente, em pacientes com IC sob tratamento farmacológico, sobre os quais não temos informações a respeito das drogas utilizadas.

É importante ressaltar que a estimulação do lóbulo da orelha (um local inervado pelo nervo auricular maior) é comumente utilizada para simulações de estimulação ( *sham* ), em estudos controlados. E foi essa a região escolhida no estudo citado. A RNM encefálica funcional, no entanto, demonstrou que essa região não é fisiologicamente inerte e há, portanto, necessidade de explorar locais alternativos para estimulação simulada ( *sham* ). ^21 , 22^

A literatura é escassa em publicações anatômicas e funcionais e não há consenso claro sobre os locais mais densamente inervados pelo ramo auricular do vago. As regiões cerebrais ativadas pela ENV auricular dependem de parâmetros específicos do estimulador, também ainda não bem definidos. Os resultados dos estudos de RNM funcional sugerem que a concha e o tragus interno sejam os sítios mais adequados para essa intervenção. ^21 , 22^

À despeito do grande entusiasmo sobre a potencialidade da estimulação vagal auricular nas mais diversas situações clínicas, se esta terapia corresponderá às expectativas ainda é uma incógnita. ^27^ As inúmeras publicações no assunto utilizam sistemas e metodologias de estimulação diversos, o que torna sua aplicabilidade duvidosa. Questões como a duração da estimulação, o tempo de tratamento, parâmetros relacionados ao estímulo, como largura de pulso, frequência e intensidade, bem como a definição da área de aplicação ainda requerem muitos estudos experimentais e ensaios clínicos amplos, com acompanhamento de longo prazo, para a ENV transauricular tornar-se uma ferramenta terapêutica comprovadamente útil nas diversas áreas da medicina.
