# Supplementary material for: Estimulação Auricular do Nervo Vago na Insuficiência Cardíaca: Análise Crítica e Perspectivas Futuras
Source: Arq Bras Cardiol. 2023 Jun 7;120(5):e20230298. [Article in Portuguese] doi: 10.36660/abc.20230298 (PMC12092030; doi:10.36660/abc.20230298)
Supplement: Translation

# Translation: Auricular Vagus Nerve Stimulation in Heart Failure: Critical Analysis and Future Perspectives

**Authors:** Denise Tessariol Hachul

**Affiliations:** 1 Brazil Instituto do Coração do Hospital das Clínicas da Faculdade de Medicina da Universidade de São Paulo, São Paulo, SP – Brazil

**Keywords:** Auricular Stimulation/methods, Vagus Nerve, Epilepsy/prevention and control, Heart Failure, Transcutaneous Electric Nerve Stimulation/methods

The term VAGUS, from the Latin, was chosen to name the cranial nerve with the most complex diversity of functions and which affects numerous physiological processes, such as autonomic, immunological, cardiovascular, gastrointestinal, respiratory and endocrine regulation.^1^

The vagus nerve is made up of 20% efferent fibers and 80% afferent fibers, which make reciprocal connections between the brain and our organs. Afferent fibers transmit sensory information upward and terminate in four nuclei located in the medulla, including the nucleus tractus solitarius (NTS). Its efferent functions include sending parasympathetic cholinergic signals from brain nuclei to target organs.^1 , 2^

Invasive vagus nerve stimulation (VNS) has been established as a therapy for refractory epilepsies for approximately 20 years. Because it has a single entrance to the brain, its electrostimulation promotes modulating access to certain brain subcortical areas.^3 - 6^

In the 1930s, it was known that VNS caused changes in the electroencephalogram (EEG). In 1988, the first device was implanted and, in 1997, approved by the FDA for the treatment of refractory epilepsy, after demonstrations of reduction in the number and severity of seizures, in patients unresponsive to pharmacological and surgical treatments.^5 , 6^

The so-called vagal “stimulator” has been implanted through a minimally invasive surgery, with the insertion of an electrode around the left cervical vagus nerve and a current generator in the infraclavicular region, similarly to an artificial cardiac pacemaker.^4^Stimulation on the left side left is preferred as it reduces the risk of bradycardia or asystole, which can be induced by stimulation of the right cervical vagus.

VNS can cause a range of side effects: from hoarseness, dysphagia, and local pain (due to its proximity to the glossopharyngeal nerve), to sudden atrioventricular blocks, due to its efferent effect on the sinus and atrioventricular node, but technical improvement and parameter adjustments of stimulation made the risk/benefit balance worthwhile, especially by reducing the mortality rate in patients with intractable epilepsy.^4 - 6^

Despite numerous studies demonstrating its effectiveness, the mechanism by which VNS controls epilepsy has not yet been fully understood. There are hypotheses that it acts by reducing brain excitability^1^and by its anti-inflammatory action, as the role of inflammation in the occurrence of epileptic seizures has recently been demonstrated.

After the success of therapy for epilepsy, the effects of VNS began to be studied more and clinical applications began to be tried for many other conditions,^7 - 12^such as migraine, obesity, depression, inflammatory bowel diseases and heart failure (HF).^14 , 15^

In the context of HF, still using implantable devices, two important clinical studies were carried out. NECTAR-HF demonstrated a statistically significant improvement in quality of life and functional class (NYHA) in the group submitted to the intervention, but not in echocardiographic parameters and autonomic tone. In INOVATE-HF, patients who received an implantable device or conventional medical treatment showed no difference in primary outcomes (death from all causes, worsening heart failure, and left ventricular end-systolic volume). But there was an improvement in quality of life and in the result of the 6-minute walk test in the stimulated group.^14 - 16^

Due to its invasive nature, side effects and complications inherent to the surgery, non-invasive VNS modalities began to be developed, particularly trans auricular stimulation.^1 , 2 , 17^The auricular branch of the vagus nerve can indirectly influence the cardiovascular system, via afferent pathways that reach the nucleus tractus solitarius (NTS), which neurons project to cardioinhibitory efferent central vagal neurons. These, in turn, propagate parasympathetic activity to the sinus node and AV node. The NTS, through other pathways, also inhibits excitatory impulses to sympathetic preganglionic neurons in the spinal cord. This inhibition decreases sympathetic activity to the cardiovascular system.^18 , 19^

Although potentially effective and less invasive, proposed atrial pacing protocols and their results vary greatly.^2 , 20 , 21^The region encompasses other auricular nerves that may be inadvertently stimulated. Stimulation of different auricular nerves can induce different cerebral and peripheral effects, which affect the reproducibility of the method.^2 , 22^Multiple reflexes can be triggered, such as the Arnold cough reflex and tearing, headache, local skin irritation, dizziness and even syncope.

The article published by *Arquivos Brasileiros de Cardiologia* ^23^prospectively evaluated 2 groups of patients with compensated HF. In the intervention group, they used a transcutaneous electrode on the superior concha and the other on the right earlobe, with the aim of stimulating terminations of the vagus, but also of the great auricular nerve of the lobe, for technical ease and standardization of the protocol. In the sham group, both electrodes were placed on the right earlobe. The intervention time was empirically determined, as well as the duration of treatment.

The intervention group was older and had a higher rMSSD index, and the sham group had a better quality of life index and better NYHA functional class at the time of recruitment. In both groups there was an improvement in performance in the 6mWT. In the intervention group, the improvement was statistically significant, but even so, the performance was inferior to that of the sham group.

An improvement in quality of life was observed in the intervention group, with no difference in the control group, whose quality of life was higher before the intervention.

Heart rate variability (HRV) is a method that has been used for years to assess vagal tonus, considered cardioprotective, associated with other clinical and laboratory variables.^24 - 26^It is noted that the rMSSD values were higher before the interventions in the sham group and no significant difference was observed between the groups after the treatment. The only parameter that improved after stimulation was the SDNN. However, it is difficult to assess the value of a single HRV parameter in patients with HF under pharmacological treatment, for whom we have no information regarding the drugs used.

It is important to note that stimulation of the earlobe (a site innervated by the greater auricular nerve) is commonly used for sham stimulation in controlled studies. And this was the region chosen in this specific study. Functional brain MRI, however, demonstrated that this region is not physiologically inert and there is, therefore, a need to explore alternative sites for sham stimulation.^21 , 22^

The literature is scarce in anatomical and functional publications and there is no clear consensus on the sites most densely innervated by the auricular branch of the vagus nerve. The brain regions activated by atrial VNS depend on specific parameters of the stimulator, which are also not yet well defined. The results of functional MRI studies suggest that the concha and internal tragus are the most suitable sites for this intervention.^21 , 22^

Despite the great enthusiasm about the potential of atrial vagal stimulation in the most diverse clinical situations, whether this therapy will meet expectations is still unknown.^27^The numerous publications on the subject use different stimulation systems and methodologies, which makes its applicability doubtful.

Issues such as stimulation duration, treatment time, stimulus-related parameters such as pulse width, frequency, and intensity, as well as the definition of the area of application still require many experimental studies and clinical trials, with long-term follow-up, for trans auricular VNS to become a proven useful therapeutic tool in HF and other several areas of medicine.
